# Long Term Association between Serum 25-Hydroxyvitamin D and Mortality in a Cohort of 4379 Men

**DOI:** 10.1371/journal.pone.0151441

**Published:** 2016-03-17

**Authors:** Haakon E. Meyer, Nathalie C. Støer, Sven O. Samuelsen, Rune Blomhoff, Trude E. Robsahm, Magritt Brustad, Edward L. Giovannucci, Tone Bjørge

**Affiliations:** 1 Department of Community Medicine, Faculty of Medicine, University of Oslo, Oslo, Norway; 2 Division of Epidemiology, Norwegian Institute of Public Health, Oslo, Norway; 3 Department of Nutrition, Harvard T.H. Chan School of Public Health, Boston, Massachusetts, United States of America; 4 Department of Mathematics, Faculty of Mathematics and Natural Sciences, University of Oslo, Oslo, Norway; 5 Department of Medical Epidemiology and Biostatistics, Karolinska Institutet, Stockholm, Sweden; 6 Department of Nutrition, Institute of Basic Medical Sciences, University of Oslo, Oslo, Norway; 7 Division of Cancer, Surgery and Transplantation, Oslo University Hospital, Oslo, Norway; 8 The Cancer Registry of Norway, Institute of Population-based Cancer Research, Oslo, Norway; 9 Department of Community Medicine, UIT The Artic University of Norway, Tromsø, Norway; 10 Department of Epidemiology, Harvard T.H. Chan School of Public Health, Boston, Massachusetts, United States of America; 11 Department of Global Public Health and Primary Care, University of Bergen, Bergen, Norway; University of Alabama at Birmingham, UNITED STATES

## Abstract

**Objective:**

A number of observational studies have shown an inverse association between circulating 25-hydroxyvitamin D and total mortality, but a reverse J-shaped association has also been reported. In a large nested case-control study, serum-25-hydroxyvitamin D (s-25(OH)D) was positively associated with incident prostate cancer. Based on the same study population, the primary aim of the present study was to investigate the association between s-25(OH)D and total mortality.

**Methods:**

Men participating in population based health screenings during 1981–1991 and enrolled in a nested case-control study were followed throughout 2007 with respect to all-cause and cause-specific mortality. Hazard ratios (HR) with 95% confidence intervals (CI) were calculated using Cox proportional hazards regression.

**Results:**

In men with prostate cancer (n = 2282), there was a significant inverse association between s-25(OH)D and total mortality after controlling for potential confounders (HR = 1.25 (95% CI 1.05–1.50), s-25(OH)D <50 nmol/l versus s-25(OH)D ≥50 nmol/l). The corresponding figure among controls (n = 2147) was HR = 1.15 (95% CI 0.88–1.50) and in the total study population HR = 1.19 (95% CI 1.03–1.38). For cause-specific deaths, we found no significant associations.

**Conclusions:**

In this study population, s-25(OH)D was inversely associated with total mortality during more than two decades of follow-up, despite, as previous reported, high s-25(OH)D was associated with increased risk of prostate cancer.

## Introduction

A number of observational studies have assessed the association between circulating 25-hydroxyvitamin D and mortality, indicating a potential beneficial role of vitamin D. Most studies have reported increased mortality in individuals low in vitamin D, and according to a recent analysis of eight cohort studies [1], low serum 25-hydroxyvitamin D (s-25(OH)D) was related to increased all-cause and cardiovascular mortality. In addition, low s-25(OH)D was related to cancer mortality in subjects with a history of cancer but not in subjects without a history of cancer, suggesting that vitamin D influences the prognosis of cancer.

A Danish study recently showed that both low plasma 25-hydroxyvitamin D and genetic variants, affecting the concentration of 25-hydroxyvitamin D, were associated with increased total mortality [2]. The polymorphisms were also associated with cancer mortality, non-cancer non-CVD mortality, but not with CVD mortality.

In addition, randomized controlled trials have reported an effect of vitamin D supplementation on mortality. Most of these studies were set up to study fractures, and some of them co-supplemented with calcium. According to a recent Cochrane review, vitamin D_3_ supplementation slightly decreases all-cause mortality (RR 0.94 (95% CI 0.91 to 0.98)) [3]. However, the result was not considered robust enough to recommend widespread supplementation, and there are several large-scale trials underway to study if vitamin D supplementation can reduce mortality [4].

Vitamin D might affect mortality through different mechanisms. The vitamin D receptor and the enzyme 1-α-hydroxylase required for activation of vitamin D are present in many organs and tissue [5]. The human genome has many binding sites for vitamin D, suggesting regulation of many genes [6]. Vitamin D has also been positively associated with leucocyte telomere length, a predictor of longevity and age-related diseases [7].

On the other hand, some cohort studies have reported a reverse J-shaped association also indicating increased mortality associated with high concentration of vitamin D [5, 8–10].

There are several concerns when assessing the effect of vitamin D in observational studies. High s-25(OH)D might be an indicator of a general healthy lifestyle. Diseases might directly or indirectly cause low vitamin D-status with the possibility of reverse causation [11].

As previously reported, high s-25(OH)D was associated with an increased risk of prostate cancer in a large Norwegian nested case-control study [12]. Interestingly, only subjects with samples collected during summer and autumn showed this association, while no relation was observed in subjects with serum collected during winter or spring.

In the present study, based on the same study population, the primary aim was to investigate the association between s-25(OH)D and total mortality and secondarily between s-25(OH)D and casus-specific mortality. Thus, we ask the question, did the increased risk of prostate cancer associated with high concentration of s-25(OH)D translate into increased mortality, or was the opposite the case?

## Material and Methods

As previously described[12], data from population based standardized cardiovascular health screenings carried out in 17 of Norway’s 19 counties during 1981–1991 by the National Health Screening Service (later Norwegian Institute of Public Health) were used, except for the capital city of Oslo where the local health authority conducted the study. All men and women in selected birth cohorts residing in the counties were invited to participate, and in men the median participation rate was 71% (range: 55–88%). Measurement of blood pressure, height and weight were included in the health examination, and body mass index (BMI) (kg/m^2^) was calculated. A non-fasting blood sample was drawn for analyses of blood lipids, and residual serum was stored at –25°C in the JANUS serum bank (http://www.kreftregisteret.no/en/Research/Janus-SerumBank/). As described earlier, total s-25(OH)D, including s-25(OH)D_3_ and s-25(OH)D_2_, was determined at Vitas, Oslo, Norway, by HPLC atmospheric pressure chemical ionization mass spectrometry (MS) [12].

The participants also filled in a questionnaire including history and symptoms of cardiovascular disease and questions on physical activity and smoking history. Physical activity during leisure time was assessed by a four graded question. Information on education was received from the Norwegian National Education Database at Statistics Norway.

Data on 122,083 men participating in the health screenings were linked to the Cancer Registry of Norway. It was required that all cancer cases had donated serum at least one year before diagnosis. In all, 2282 men who were diagnosed with prostate cancer after screening and throughout 2006 were included.

In the original data setup for the matched case-control study [12], one control was selected for each case matched for age at serum sampling (±6 months), date of serum sampling (±2 months), and health examination. Controls were alive and free from cancer at the time of diagnosis of the corresponding case. As controls could randomly be selected several times according to the protocol, we obtained 2147 unique controls. Fifty persons were excluded due to missing data. Thus, the final dataset included 4379 men (2259 cases and 2120 controls).

The participants in the original case-control study have been followed with respect to total and cause-specific mortality by linkage to the National Population Registry and the Cause of Death Registry. Follow-up time was calculated from the index date, that is the date of prostate cancer diagnosis for cases and the diagnosis date of the matched case for the controls, to the date of death or throughout 2007.

### Statistics

Descriptive characteristics of the study population are presented as means with standard deviation (SD) and frequencies (%), according to s-25(OH)D.

Hazard ratios (HR) with 95% confidence intervals (CI) were calculated using Cox proportional hazards regression models with follow-up from index date. The data was analysed not taking the original matching into account. Instead, adjustment was made to account for the matching (case/control status (applies only for the complete dataset), age, month of serum sampling, and health examination). In addition, we adjusted for education (primary, secondary, and university or college), BMI, smoking (current cigarette smoking yes/no) and physical activity during leisure time (sedentary, moderate, intermediate, and intensive). To produce spline curves, s-25(OH)D was included as restricted cubic splines with five knots.

In an alternative analysis, we analysed the complete dataset (n = 4379) as a matched cohort study utilizing stratified Cox proportional hazards regression models. The results were similar (S1 Appendix), but the statistical power was lower. We therefore present the data from the un-stratified Cox model.

In addition, we performed a competing risk analysis in the prostate cancer cases by the stcrreg command in STATA in order to estimate the cumulative mortality in groups of s-25(OH)D when considering the two competing events: deaths from prostate cancer (underlying cause, n = 381) and death from all other causes (n = 228).

For comparison we also re-analyzed the association between s-25(OH)D and prostate cancer by a weighted Cox-regression [13, 14] adjusted for age, month of blood sampling, examination and education to obtain comparable hazard ratios. The weights were estimated with logistic regression and the approach enables us to break the matching between cases and controls.

Data were analysed by IBM SPSS 22, STATA 13.1 and R (R Core Team. R: A Language and Environment for Statistical Computing. http://www.R-project.org/).

The study was approved by the Regional Committee for Medical Research Ethics of Western Norway.

## Results

During follow-up, 609 of 2259 men with prostate cancer (27%) and 267 of 2120 men without prostate cancer (12.6%) died. Mean time from blood sample collection to the end of follow-up was 21.1 (SD±4.0) years. Mean time from blood sample collection to death was 16.8 (SD±5.6) years and only 2.3% of the deaths occurred within 5 years and 12% of the deaths occurred within 10 years from blood collection. Mean follow-up time from the time of diagnosis (index date) was 4.7 (SD±3.4) years.

Baseline concentration of s-25(OH)D was inversely associated with BMI and being a current cigarette smoker, positively associated with physical activity and education whereas there was no clear association with height and total serum cholesterol (Table 1)

**Table 1:** Characteristics of the study population at baseline by s-25(OH)D concentration.

	s-25(OH)D (nmol/l)				
	< 30	30–49	50–69	70–89	≥90
N	160	1141	1561	1023	494
s-25(OH)D (nmol/l)^a^	25.5 (3.9)	41.7 (5.4)	59.7 (5.7)	78.5 (5.6)	105.6 (16.5)
Age^a^	47.1 (8.8)	47.6 (8.6)	48.5 (9.4)	48.6 (9.4)	48.0 (9.1)
BMI (kg/m2) ^a^	26.0 (3.7)	25.9 (3.1)	25.7 (2.9)	25.3 (2.9)	24.7 (2.6)
Height (m) ^a^	1.77 (.07)	1.77 (.07)	1.77 (.07)	1.77 (.06)	1.77 (.07)
Leisure time activity (%)					
Sedentary	28%	21%	15%	14%	11%
Moderate	56%	55%	55%	53%	52%
Intermediate	15%	23%	28%	30%	33%
Intensive	1%	1%	2%	3%	4%
Current cigarette smoker (%)	49%	41%	31%	29%	28%
Total serum cholesterol (mmol/l) ^a^	6.0 (1.1)	6.1 (1.2)	6.1 (1.1)	6.1 (1.1)	6.0 (1.1)
Education (%)					
Primary	40%	38%	32%	36%	30%
Secondary	46%	47%	48%	43%	48%
University or college	14%	15%	20%	21%	22%

^a^ Mean±SD

**Fig 1:**
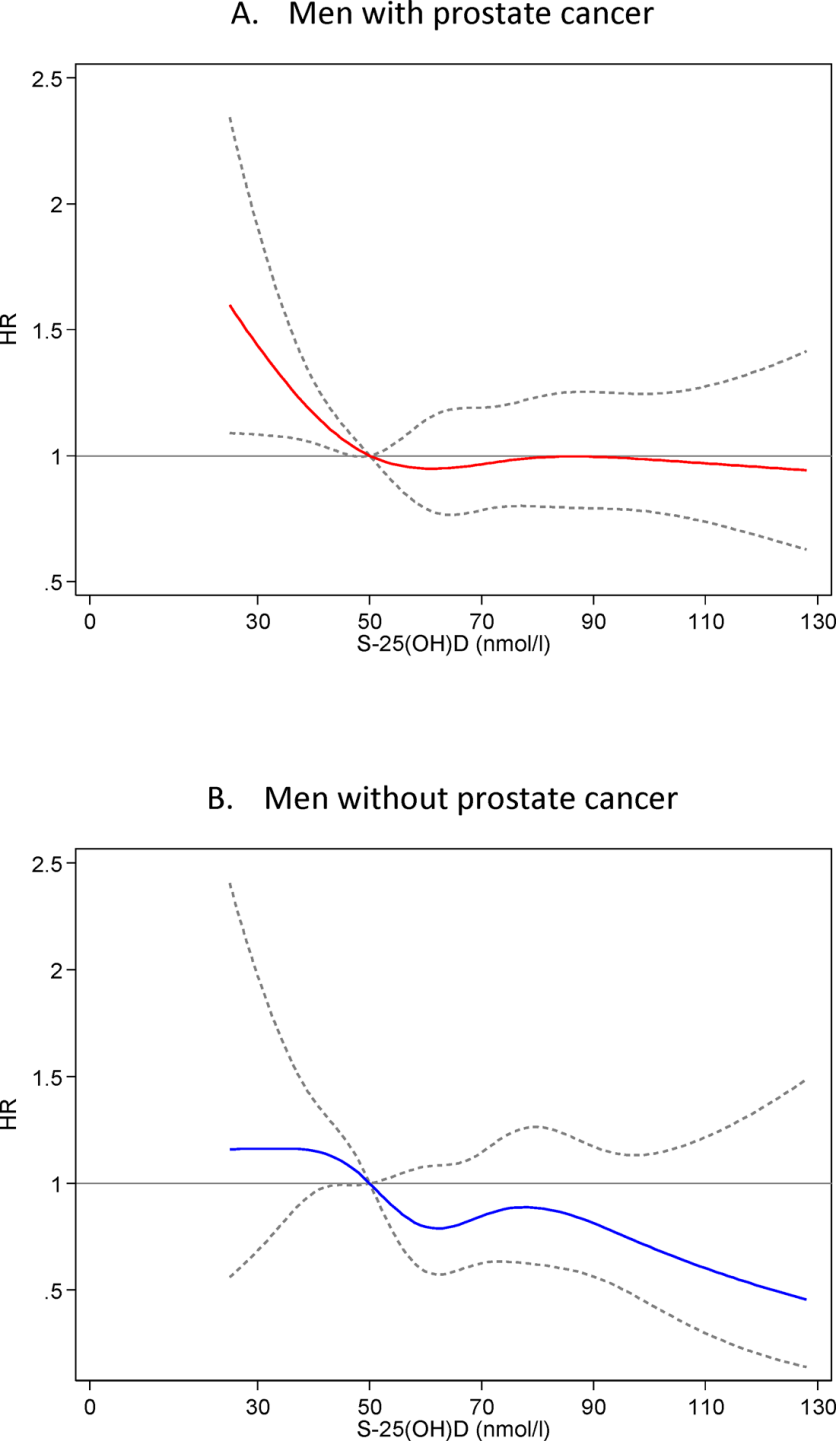
Hazard ratios (solid lines) with 95% confidence intervals (dashed lines) for all-cause mortality across the distribution of 25(OH)D in (A) men with prostate cancer and in (B) men without prostate cancer. Adjusted for age, month of blood sampling, examination, physical activity, BMI, smoking and education. 25(OH)D was included as restricted cubic splines with five knots.

As can be seen in Table 2, there was an inverse association between baseline s-25(OH)D concentration and total mortality both in men with and without prostate cancer. Further adjustment for BMI, smoking, physical activity and education attenuated the associations moderately, and the association remained significant in men with prostate cancer only. According to spline analyses (Fig 1), the curves showing the association between s-25(OH)D and total mortality were relatively flat at concentration higher than 60–70 nmol/l, and there was no indication of increased risk in those with the highest concentration of s-25(OH)D. In the analyses restricted to men with prostate cancer, the HR for total mortality in men with s-25(OH)D < 50 nmol/l was HR = 1.25 (95% CI 1.05–1.50) in the fully adjusted model, compared to men with higher s-25(OH)D. The corresponding HR in the controls was 1.15 (95% CI 0.88–1.50) and in the total study population the HR was 1.19 (95% CI 1.03–1.38).

**Table 2:** Hazard ratio (HR) and 95% confidence intervals (95% CI) for total mortality by concentration of s-25(OH)D.

25(OH)D (nmol/l)	n	Deaths, n (%)	HR (95% CI)^a^	HR (95% CI)^b^
	***Men with prostate cancer***			
< 30	74	24 (32.4)	1.60 (1.04, 2.46)	1.43 (0.93, 2.22)
30–49	571	172 (30.1)	1.32 (1.07, 1.63)	1.28 (1.03, 1.58)
50–69	781	200 (25.6)	1.00 (ref)	1.00 (ref)
70–89	563	148 (26.3)	1.10 (0.89, 1.37)	1.10 (0.89, 1.37)
≥ 90	270	65 (24.1)	0.95 (0.71, 1.26)	0.97 (0.73, 1.30)
*P*_*linear trend*_			*0*.*028*	*0*.*095*
HR per 20 nmol/l decrease^c^			1.09 (1.01, 1.18)	1.07 (0.99, 1.16)
	***Men without prostate cancer***			
< 30	86	8 (9.3)	0.92 (0.44, 1.93)	0.90 (0.42, 1.90)
30–49	570	81 (14.2)	1.23 (0.91, 1.66)	1.13 (0.83, 1.52)
50–69	780	99 (12.7)	1.00 (ref)	1.00 (ref)
70–89	460	59 (12.8)	0.93 (0.67, 1.29)	0.95 (0.68, 1.32)
≥ 90	224	20 (8.9)	0.82 (0.51, 1.34)	0.82 (0.50, 1.34)
*P*_*linear trend*_			*0*.*025*	*0*.*083*
HR per 20 nmol/l decrease^c^			1.16 (1.02, 1.32)	1.12 (0.99, 1.28)

^a^ HR estimated by Cox regression adjusted for age, month of blood sampling and examination

^b^ Additionally adjusted for physical activity, BMI, smoking and education

^c^ Per 20 nmol/l decrease in s-25(OH)D entered as a continuous variable

In those with prostate cancer, we also analyzed the data according to underlying causes of death, grouping them into deaths from cancer (cancer deaths, n = 460), cardiovascular diseases (CVD deaths, n = 96), and all other causes (non-CVD, non-cancer deaths, n = 53). No significant associations were found although the estimates suggested a moderate increased mortality in those with low vitamin D status (Table 3). For deaths from prostate cancer (underlying cause, n = 381), the HR per 20 nmol/l decrease in s-25(OH)D was 1.06 (95% CI 0.96–1.17), and the HR for those having s-25(OH)D concentration < 50 nmol/l compared to ≥ 50 nmol/l was 1.15 (0.91–1.45).

**Table 3:** Hazard ratio (HR) and 95% confidence interval (95% CI) for cause specifics deaths in patients with prostate cancer.

s-25(OH)D, (nmol/l)	n	Deaths, n (%)	HR (95% CI)^a^
	***Cancer deaths***		
< 50	645	146 (22.6)	1.20 (0.97, 1.48)
≥ 50	1614	314 (19.5)	1.00 (reference)
HR per 20 nmol/l decrease^b^			1.06 (0.97, 1.16)
	***CVD deaths***		
< 50	645	30 (4.7)	1.40 (0.88, 2.23)
≥ 50	1614	66 (4.1)	1.00 (reference)
HR per 20 nmol/l decrease^b^			1.06 (0.87, 1.28)
	***Non-CVD non-cancer deaths***		
< 50	645	20 (3.1)	1.52 (0.83, 2.78)
≥ 50	1614	33 (2.0)	1.00 (reference)
HR per 20 nmol/l decrease^b^			1.18 (0.89, 1.56)

^a^ HR estimated by Cox regression adjusted for age, month of blood sampling, examination, physical activity, BMI, smoking and education

^b^ Per 20 nmol/l decrease in s-25(OH)D entered as a continuous variable

In analyses restricted to blood samples collected during winter/spring in those with prostate cancer, the HR for prostate cancer deaths was 1.15 (95% CI 0.97–1.35) per 20 nmol/l decrease in s-25(OH)D. The corresponding result for men with blood samples collected during summer/autumn was 1.03 (95% CI 0.90–1.17).

**Fig 2:**
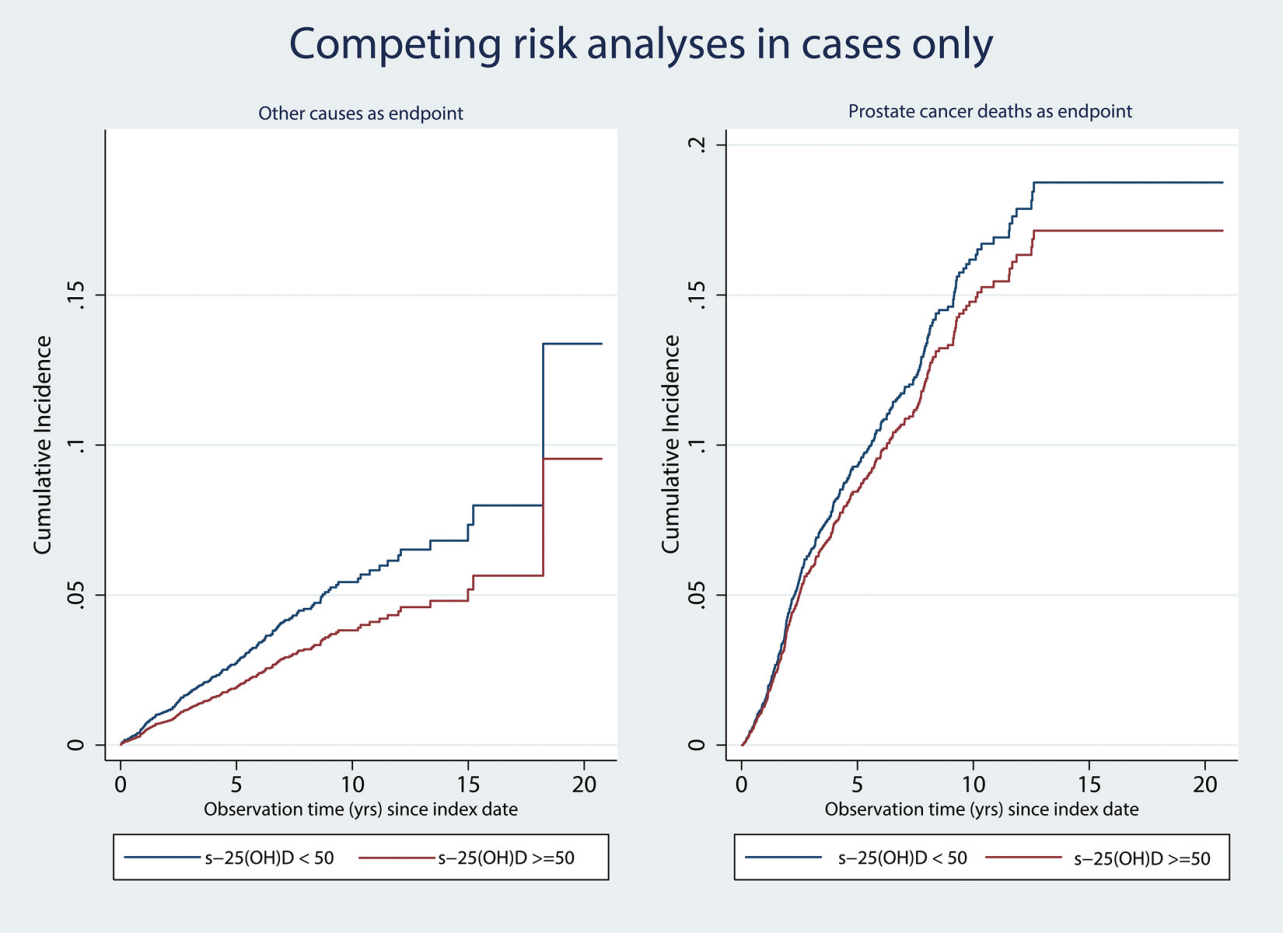
Cumulative mortality from other causes than prostate cancer (A) and cumulative mortality from prostate cancer (B), in men with a prostate cancer diagnosis (n = 2259) according to strata of 25(OH)D concentration. Adjusted for age, month of blood sampling, examination, physical activity, BMI, smoking and education. Death from prostate cancer (n = 381) and death from all other causes (n = 228) was set as competing events in panel A and B, respectively.

Results from the competing risk analyses, restricted to men with prostate cancer, are shown in Fig 2. The cumulative incidence of deaths from other causes was higher in those with s-25(OH)D < 50nmol/l compared to those with s-25(OH)D ≥ 50 nmol/l (p = 0.015), whereas the corresponding difference was not significant in the analysis with cumulative incidence of death from prostate cancer (p = 0.41).

In analyses restricted to men without prostate cancer, the HR per 20 nmol/l decrease in s-25(OH)D was 0.94 (95% CI 0.75–1.18) for cancer deaths (n = 69), 1.20 (95% CI 0.98–1.47) for CVD deaths (n = 117), and 1.23 (95% CI 0.97–1.57) for non-CVD non-cancer deaths (n = 81).

**Fig 3:**
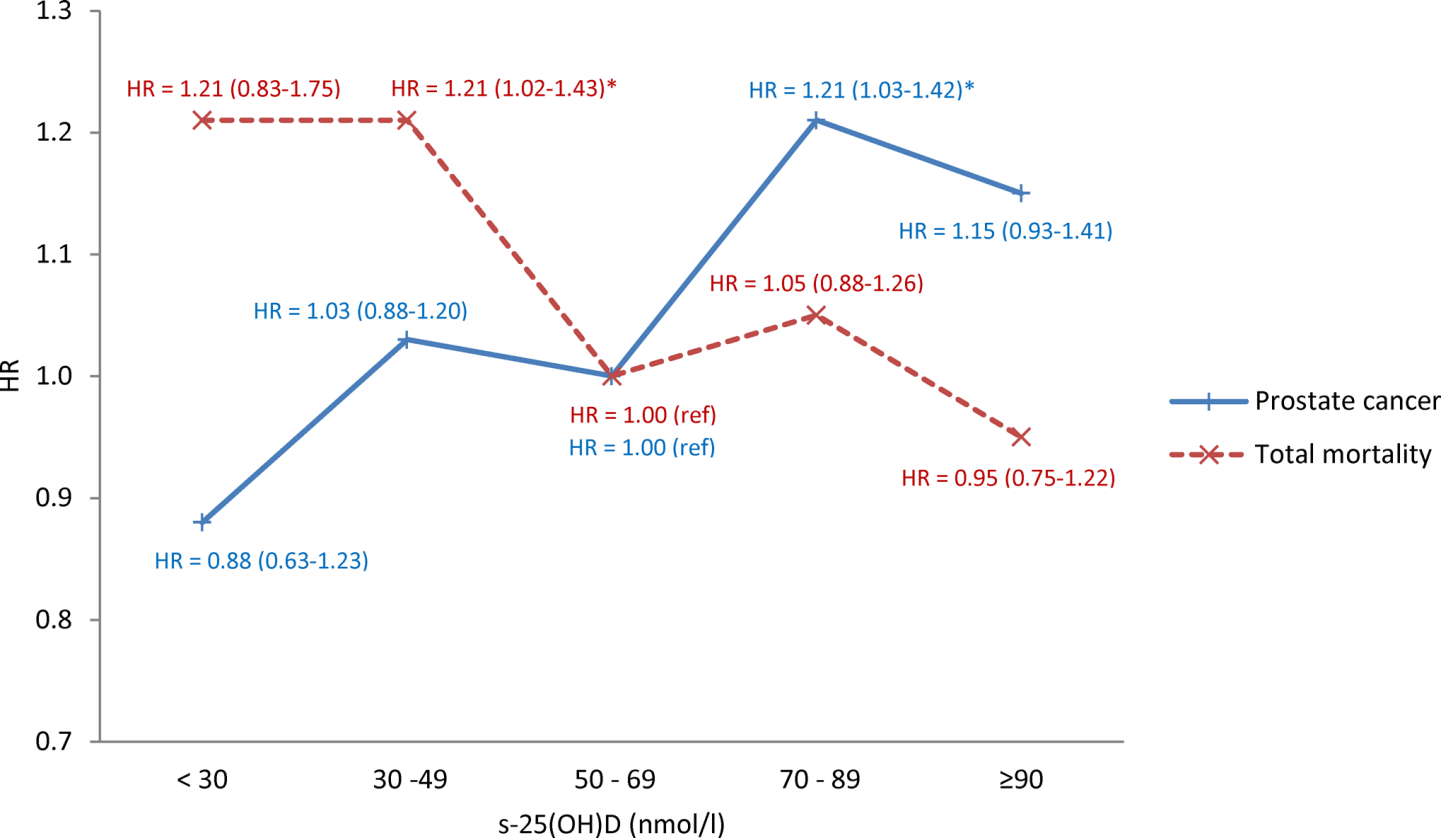
Hazard Ratios (HR) with 95% confidence intervals for total mortality (red line) and incident prostate cancer (blue line) by category of s-25(OH)D. Separate analyses were performed for the two endpoints. 25(OH)D 50–69 nmol/l is the reference category. In the total mortality analysis, adjustment was made for age, case status, month of blood sampling, examination, physical activity, BMI, smoking and education. In the prostate cancer incidence analysis adjustment was made for age, month of blood sampling, examination and education.

Analysing the prostate cancer cases and controls groups together, there was no interaction between having prostate cancer or not and s-25(OH)D (p_*interaction*_ = 0.39) on mortality. As the HR’s for death in the different categories of s-25(OH)D was rather similar in men with and without prostate cancer (except for those with s-25(OH)D < 30 nmol/l), a combined analysis is shown in Fig 3: The overall result in this study population was an inverse association between s-25(OH)D and all-cause mortality. In addition we reproduced the previously reported finding [12] of an increasing risk of incidence of prostate cancer by increasing concentration of s-25(OH)D.

## Discussion

In this study population, low s-25(OH)D was associated with increased risk of total mortality during more than two decades of follow-up from the blood sample collection, whereas as previous reported, high s-25(OH)D was associated with increased risk of incident prostate cancer [12].

The inverse association between s-25(OH)D and total mortality is in agreement with numerous other studies [1, 2, 15–17]. The association was, however, not strong with a roughly 10% decreased mortality by a 20 nmol/l increase in s-25(OH)D, comparable in magnitude to what was found in a large British cohort study [16] and meta-analyses of prospective cohort studies [15, 17]. On the other hand, if there is a causal effect of vitamin D on mortality, there is reason to believe that this effect is not very large. According to the Cochrane review on this topic, supplementation with vitamin D_3_ modestly decreases all-cause mortality with 6% (RR = 0.94 (95% CI 0.91 to 0.98)) [3]. On the other hand, some observational studies have reported increased mortality in those with the highest concentration of 25(OH)D [8–10]. We did not find any indication of this in spite of increased incidence of prostate cancer by increasing s-25(OH)D. However, few men in our study had very high concentration of s-25(OH)D (1.1% had concentration ≥ 125 nmol/l and 6% had concentration ≥ 100 nmol/l). In those with blood collected during summer (June through August), 12.9% had concentrations > = 100 nmol/l, indicating that sun exposure was an important determinant for high s-25(OH)D in the study population.

Some other studies have also reported increased risk of incident prostate cancer at high concentrations of circulating 25-hydroxyvitamin D [18–21]. Although our study had limited statistical power to study cause specific mortality, it is reassuring that the increased risk of incident prostate cancer was not translated into increased risk of fatal prostate cancer as a non-significant inverse association between increasing s-25(OH)D and fatal prostate cancer was found. Also in an alternative analyses of the total study population, there was no significant association between s-25(OH)D and death from prostate cancer [13]. In a previous Norwegian study of 160 patients with prostate cancer, s-25(OH)D in blood samples collected close to the time of diagnosis (±3 months) was inversely associated with death from prostate cancer [22]. One American study also found that plasma 25-hydroxyvitamin D was inversely and statistically significantly related to fatal prostate cancer [23], whereas this was not confirmed in an extended analysis in the Breast and Prostate Cancer Cohort Consortium (518 fatal cases) with an odds ratio of 0.86 (95% CI 0.65–1,14) in the highest compared to the lowest quartile of circulating 25-hydroxyvitamin D [24]. This is in line with our results (381 fatal cases) with a RR of 0.93 (95% CI 0.69–1.23) in the highest compared to the lowest quartile of s-25(OH)D.

In general, the effect of vitamin D might be stronger for mortality of cancer than for incidence of cancer. In a recent meta-analysis of randomized controlled trials, no effect of vitamin D supplementation was found on cancer incidence (400–1100 IU per day over 2–7 years), whereas a 12 percent reduction in total cancer mortality was reported [25].

The mechanisms for an effect of vitamin D on total mortality are not clear but could act via different pathways. Many genes are regulated by vitamin D [6], and many organ and tissues have vitamin D receptors and the enzyme 1-α-hydroxylase required for activation of vitamin D [5]. In addition to vitamin D’s effects on calcium and bone metabolism, vitamin D might have many extra-skeletal effects, including an influence on cell proliferation and differentiation, the immune system, the cardiovascular system, metabolism and the neurological system. It is thus biologically plausible that vitamin D might positively influence on a number of diseases, and this seems to be supported by observational studies [5]. In a recent meta-analysis of primary prevention cohorts, high circulating 25-hydroxyvitamin D was inversely associated both with cardiovascular deaths, cancer deaths and deaths due to other causes [15]. This is somewhat in line with the cause-specific analyses in our study, although these results were not statistically significant and had wide confidence intervals.

A limitation of our study was that only one measurement of s-25(OH)D from each participant was available, but as discussed in the previous publication, the correlation coefficient between two measurements up to 14 years apart have been reported to be about 0.5 [12]. Another limitation was the limited statistical power to study cause-specific mortality. In the prostate cancer cases, no Gleason grading was available. As discussed in the first paper [12], increased detection of prostate cancer in those with high s-25(OH)D is probably not the case in this study population as adjustment for education had very little impact on the estimates and there was no association between s-25(OH)D and localized cancer. Although we adjusted for important confounders, residual confounding cannot be excluded. Strength of this study was a very long follow-up with few of the deaths occurring the first years after blood sample collection, reducing the risk of reverse causation. The statistical power to study total mortality was good. The participants were recruited from the general Norwegian population, but were restricted to men with prostate cancer and their matched controls without cancer at the time of diagnosis of their counterpart.

In conclusion, in this prospective study, low concentration of s-25(OH)D was associated with increased mortality during more than two decades of follow-up, and as previously reported, it was associated with decreased incidence of prostate cancer.

## Supporting Information

S1 Appendix (PDF)

## References

[1] Schottker B, Jorde R, Peasey A, Thorand B, Jansen EH, Groot L, et al Vitamin D and mortality: meta-analysis of individual participant data from a large consortium of cohort studies from Europe and the United States. Bmj. 2014;348:g3656 Epub 2014/06/19. 10.1136/bmj.g3656 ; PubMed Central PMCID: PMCPmc4061380. 24938302 PMC4061380

[2] Afzal S, Brondum-Jacobsen P, Bojesen SE, Nordestgaard BG. Genetically low vitamin D concentrations and increased mortality: mendelian randomisation analysis in three large cohorts. Bmj. 2014;349:g6330 Epub 2014/11/20. 10.1136/bmj.g6330 ; PubMed Central PMCID: PMCPmc4238742. 25406188 PMC4238742

[3] Bjelakovic G, Gluud LL, Nikolova D, Whitfield K, Krstic G, Wetterslev J, et al Vitamin D supplementation for prevention of cancer in adults. The Cochrane database of systematic reviews. 2014;6:Cd007469. Epub 2014/06/24. 10.1002/14651858.CD007469.pub2 . 24953955 PMC11285304

[4] Meyer HE, Holvik K, Lips P. Should vitamin D supplements be recommended to prevent chronic diseases? Bmj. 2015;350:h321 Epub 2015/01/31. 10.1136/bmj.h321 . 25633148

[5] Bouillon R, Van Schoor NM, Gielen E, Boonen S, Mathieu C, Vanderschueren D, et al Optimal vitamin D status: a critical analysis on the basis of evidence-based medicine. The Journal of clinical endocrinology and metabolism. 2013;98(8):E1283–304. Epub 2013/08/08. 10.1210/jc.2013-1195 . 23922354

[6] Ramagopalan SV, Heger A, Berlanga AJ, Maugeri NJ, Lincoln MR, Burrell A, et al A ChIP-seq defined genome-wide map of vitamin D receptor binding: associations with disease and evolution. Genome Res. 2010;20(10):1352–60. Epub 2010/08/26. 10.1101/gr.107920.110 20736230 PMC2945184

[7] Richards JB, Valdes AM, Gardner JP, Paximadas D, Kimura M, Nessa A, et al Higher serum vitamin D concentrations are associated with longer leukocyte telomere length in women. The American journal of clinical nutrition. 2007;86(5):1420–5. Epub 2007/11/10. 17991655 10.1093/ajcn/86.5.1420 PMC2196219

[8] Sempos CT, Durazo-Arvizu RA, Dawson-Hughes B, Yetley EA, Looker AC, Schleicher RL, et al Is there a reverse J-shaped association between 25-hydroxyvitamin D and all-cause mortality? Results from the U.S. nationally representative NHANES. The Journal of clinical endocrinology and metabolism. 2013;98(7):3001–9. Epub 2013/05/15. 10.1210/jc.2013-1333 ; PubMed Central PMCID: PMCPmc3701270. 23666975 PMC3701270

[9] Durup D, Jorgensen HL, Christensen J, Tjonneland A, Olsen A, Halkjaer J, et al A Reverse J-Shaped Association Between Serum 25-Hydroxyvitamin D and Cardiovascular Disease Mortality: The CopD Study. The Journal of clinical endocrinology and metabolism. 2015;100(6):2339–46. Epub 2015/02/25. 10.1210/jc.2014-4551 . 25710567

[10] Michaelsson K, Baron JA, Snellman G, Gedeborg R, Byberg L, Sundstrom J, et al Plasma vitamin D and mortality in older men: a community-based prospective cohort study. The American journal of clinical nutrition. 2010;92(4):841–8. Epub 2010/08/20. 10.3945/ajcn.2010.29749 . 20720256

[11] Reid D, Toole BJ, Knox S, Talwar D, Harten J, O'Reilly DS, et al The relation between acute changes in the systemic inflammatory response and plasma 25-hydroxyvitamin D concentrations after elective knee arthroplasty. The American journal of clinical nutrition. 2011;93(5):1006–11. Epub 2011/03/18. 10.3945/ajcn.110.008490 . 21411617

[12] Meyer HE, Robsahm TE, Bjørge T, Brustad M, Blomhoff R. Vitamin D, season, and risk of prostate cancer: a nested case-control study within Norwegian health studies. The American journal of clinical nutrition. 2013;97(1):147–54. Epub 2012/11/30. 10.3945/ajcn.112.039222 . 23193007

[13] Støer NC, Meyer HE, Samuelsen SO. Reuse of controls in nested case-control studies. Epidemiology. 2014;25(2):315–7. Epub 2014/02/04. 10.1097/ede.0000000000000057 . 24487222

[14] Samuelsen SO. A pseudolikelihood approach to analysis of nested case-control studies. Biometrika. 1997;84(2):379–94. 10.1093/biomet/84.2.379

[15] Chowdhury R, Kunutsor S, Vitezova A, Oliver-Williams C, Chowdhury S, Kiefte-de-Jong JC, et al Vitamin D and risk of cause specific death: systematic review and meta-analysis of observational cohort and randomised intervention studies. Bmj. 2014;348:g1903 Epub 2014/04/03. 10.1136/bmj.g1903 ; PubMed Central PMCID: PMCPmc3972416. 24690623 PMC3972416

[16] Khaw KT, Luben R, Wareham N. Serum 25-hydroxyvitamin D, mortality, and incident cardiovascular disease, respiratory disease, cancers, and fractures: a 13-y prospective population study. The American journal of clinical nutrition. 2014;100(5):1361–70. 10.3945/ajcn.114.086413 25332334 PMC4196486

[17] Schöttker B, Ball D, Gellert C, Brenner H. Serum 25-hydroxyvitamin D levels and overall mortality. A systematic review and meta-analysis of prospective cohort studies. Ageing Research Reviews. 2013;12(2):708–18. 10.1016/j.arr.2012.02.004. 10.1016/j.arr.2012.02.004 22343489

[18] Schenk JM, Till CA, Tangen CM, Goodman PJ, Song X, Torkko KC, et al Serum 25-Hydroxyvitamin D Concentrations and Risk of Prostate Cancer: Results from the Prostate Cancer Prevention Trial. Cancer Epidemiology Biomarkers & Prevention. 2014;23(8):1484–93. 10.1158/1055-9965.epi-13-1340 PMC4120235 25085836

[19] Tuohimaa P, Tenkanen L, Ahonen M, Lumme S, Jellum E, Hallmans G, et al Both high and low levels of blood vitamin D are associated with a higher prostate cancer risk: a longitudinal, nested case-control study in the Nordic countries. IntJCancer. 2004;108(1):104–8. 10.1002/ijc.11375 14618623

[20] Albanes D, Mondul AM, Yu K, Parisi D, Horst RL, Virtamo J, et al Serum 25-hydroxy vitamin D and prostate cancer risk in a large nested case-control study. Cancer epidemiology, biomarkers & prevention: a publication of the American Association for Cancer Research, cosponsored by the American Society of Preventive Oncology. 2011;20(9):1850–60. Epub 2011/07/26. 10.1158/1055-9965.epi-11-0403 21784952 PMC3188814

[21] Ahn J, Peters U, Albanes D, Purdue MP, Abnet CC, Chatterjee N, et al Serum vitamin D concentration and prostate cancer risk: a nested case-control study. Journal of the National Cancer Institute. 2008;100(11):796–804. Epub 2008/05/29. 10.1093/jnci/djn152 18505967 PMC3703748

[22] Tretli S, Hernes E, Berg JP, Hestvik UE, Robsahm TE. Association between serum 25(OH)D and death from prostate cancer. British journal of cancer. 2009;100(3):450–4. Epub 2009/01/22. 10.1038/sj.bjc.6604865 ; PubMed Central PMCID: PMCPmc2658536. 19156140 PMC2658536

[23] Shui IM, Mucci LA, Kraft P, Tamimi RM, Lindstrom S, Penney KL, et al Vitamin D-related genetic variation, plasma vitamin D, and risk of lethal prostate cancer: a prospective nested case-control study. Journal of the National Cancer Institute. 2012;104(9):690–9. Epub 2012/04/14. 10.1093/jnci/djs189 ; PubMed Central PMCID: PMCPmc3341310. 22499501 PMC3341310

[24] Shui IM, Mondul AM, Lindstrom S, Tsilidis KK, Travis RC, Gerke T, et al Circulating vitamin D, vitamin D-related genetic variation, and risk of fatal prostate cancer in the National Cancer Institute Breast and Prostate Cancer Cohort Consortium. Cancer. 2015 Epub 2015/03/04. 10.1002/cncr.29320 . 25731953 PMC4457645

[25] Keum N, Giovannucci E. Vitamin D supplements and cancer incidence and mortality: a meta-analysis. British journal of cancer. 2014;111(5):976–80. Epub 2014/06/12. 10.1038/bjc.2014.294 ; PubMed Central PMCID: PMCPmc4150260. 24918818 PMC4150260

