# Supplementary material for: Long Term Association between Serum 25-Hydroxyvitamin D and Mortality in a Cohort of 4379 Men
Source: PLoS One. 2016 Mar 17;11(3):e0151441. doi: 10.1371/journal.pone.0151441 (PMC4795600; doi:10.1371/journal.pone.0151441)
Supplement: S1 Appendix — (PDF) [file pone.0151441.s001.pdf]

S1 Appendix 1

Hazard ratios (HR) with 95% confidence intervals (CI) were calculated using Cox proportional hazards regression models with follow-up from index date. As described in the statistical section of material and methods, the complete dataset (cases and controls) was analyzed not taking the original matching into account. Instead, adjustment was made to account for the matching (case/control status (applies only for the complete dataset), age, month of serum sampling, and health examination).

We also analyzed the complete dataset as a matched cohort study utilizing stratified Cox proportional hazards regression models. Originally, the dataset consisted of 2216 strata with one prostate cancer case and one control in each. However, the statistical power was considerably affected by the fact that there were no deaths in many strata. We therefor collapsed similar strata by randomly selecting the first case and included all cases and controls with similar values on the matching variables into the same strata. This was repeated with the next case (not included in the first stratum) and continued until all cases and controls were included. In this way, the number of strata was reduced to 852. As the random order of the cases influenced this proses, the whole procedure was repeated, and this time the number of strata was reduced to 870.

In **Table S1** below, the results from the ordinary Cox proportional hazards regression (column A) and the stratified Cox proportional hazards regression by the first sampling (column B) and the second sampling (column C) are displayed. As can be seen, the results are similar, but the 95% confidence intervals are narrower for the ordinary Cox analysis.

Table S1. Hazard ratio (HR) and 95% confidence intervals (95% CI) for total mortality by concentration of s-25(OH)D

	**A**	**B**	**C**
25(OH)D (nmol/l)	HR (95% CI)[a]	HR (95% CI)[b]	HR (95% CI)[c]
< 30	1.35 (0.93, 1.94)	1.19 (0.77-1.82)	1.38 (0.88-2.17)
30-49	1.27 (1.07, 1.51)	1.24 (1.01-1.52)	1.26 (1.03-1.55)
50-69	1.00 (ref)	1.00 (ref)	1.00 (ref)
70-89	1.03 (0.86, 1.23)	0.93 (0.75-1.15)	0.92 (0.74-1.14)
≥ 90	0.91 (0.72, 1.17)	0.78 (0.58-1.06)	0.85 (0.63-1.14)
HR per 30 nmol/l increase[d]	*0.86 (0.78-0.95)*	*0.83 (0.73-0.93)*	*0.81 (0.72-0.92)*

[a] HR estimated by Cox adjusted for matching variables (case status, age, storage time, month of blood sampling and screening)

[b] HR estimated by stratified Cox proportional hazards regression, first sampling

[c] HR estimated by stratified Cox proportional hazards regression, second sampling

[d] Per 30 nmol/l increase in s-25(OH)D entered as a continuous variable
